# Field evaluation of a Pan-Lassa rapid diagnostic test during the 2018 Nigerian Lassa fever outbreak

**DOI:** 10.1038/s41598-020-65736-0

**Published:** 2020-05-26

**Authors:** Matthew L. Boisen, Eghosa Uyigue, John Aiyepada, Katherine J. Siddle, Lisa Oestereich, Diana K. S. Nelson, Duane J. Bush, Megan M. Rowland, Megan L. Heinrich, Philomena Eromon, Adeyemi T. Kayode, Ikponmwosa Odia, Donatus I. Adomeh, Ekene B. Muoebonam, Patience Akhilomen, Grace Okonofua, Blessing Osiemi, Omigie Omoregie, Michael Airende, Jacqueline Agbukor, Solomon Ehikhametalor, Chris Okafi Aire, Sophie Duraffour, Meike Pahlmann, Wiebke Böhm, Kayla G. Barnes, Samar Mehta, Mambu Momoh, John Demby Sandi, Augustine Goba, Onikepe A. Folarin, Ephraim Ogbaini-Emovan, Danny A. Asogun, Ekaete A. Tobin, George O. Akpede, Sylvanus A. Okogbenin, Peter O. Okokhere, Donald S. Grant, John S. Schieffelin, Pardis C. Sabeti, Stephan Günther, Christian T. Happi, Luis M. Branco, Robert F. Garry

**Affiliations:** 1 Zalgen Labs, LLC, Germantown, MD USA; 2 The African Center of Excellence for Genomics of Infectious Diseases, Redeemer’s University, Ede, Osun State Nigeria; 3 Department of Biological Sciences, College of Natural Sciences, Redeemer’s University, Ede, Osun State Nigeria; 4 Institute of Lassa Fever Research and Control, Irrua Specialist Teaching Hospital, Irrua, Edo State Nigeria; 5 The Broad Institute of Massachusetts Institute of Technology (MIT) and Harvard University, Cambridge, MA USA; 6 The Center for Systems Biology, Department of Organismic and Evolutionary Biology, Harvard University, Cambridge, MA USA; 7 Bernhard Nocht Institute for Tropical Medicine, Hamburg, Germany; 8 German Center for Infection Research (DZIF), Hamburg, Germany; 9 Department of Immunology and Infectious Diseases, Harvard T.H. Chan School of Public Health, Harvard University, Boston, MA USA; 10 Beth Israel Deaconess Medical Center, Division of Infectious Diseases, Boston, MA USA; 11 Eastern Polytechnic Institute, Kenema, Sierra Leone; 12 Viral Hemorrhagic Fever Program, Kenema Government Hospital, Kenema, Sierra Leone; 13 Ministry of Health and Sanitation, Freetown, Sierra Leone; 14 The Department of Medicine, Irrua Specialist Teaching Hospital, Irrua, Edo State Nigeria; 15 The Department of Medicine, Faculty of Clinical Sciences, Ambrose Alli University, Ekpoma, Edo State Nigeria; 16 College of Medicine and Allied Health Sciences, University of Sierra Leone, Freetown, Sierra Leone; 17 Sections of Infectious Disease, Departments of Pediatrics and Internal Medicine, School of Medicine, Tulane University, New Orleans, LA USA; 18 Harvard-MIT Health Sciences and Technology, MIT, Cambridge, MA USA; 19 Howard Hughes Medical Institute, Chevy Chase, MD USA; 20 Tulane Health Sciences Center, Tulane University, New Orleans, LA USA; 21 Tulane University, School of Medicine, Department of Microbiology and Immunology, New Orleans, LA USA

**Keywords:** Immunology, Microbiology

## Abstract

Lassa virus (LASV) is the causative agent of Lassa fever (LF), an often-fatal hemorrhagic disease. LF is endemic in Nigeria, Sierra Leone and other West African countries. Diagnosis of LASV infection is challenged by the genetic diversity of the virus, which is greatest in Nigeria. The ReLASV Pan-Lassa Antigen Rapid Test (Pan-Lassa RDT) is a point-of-care, *in vitro* diagnostic test that utilizes a mixture of polyclonal antibodies raised against recombinant nucleoproteins of representative strains from the three most prevalent LASV lineages (II, III and IV). We compared the performance of the Pan-LASV RDT to available quantitative PCR (qPCR) assays during the 2018 LF outbreak in Nigeria. For patients with acute LF (RDT positive, IgG/IgM negative) during initial screening, RDT performance was 83.3% sensitivity and 92.8% specificity when compared to composite results of two qPCR assays. 100% of samples that gave Ct values below 22 on both qPCR assays were positive on the Pan-Lassa RDT. There were significantly elevated case fatality rates and elevated liver transaminase levels in subjects whose samples were RDT positive compared to RDT negative.

## Introduction

Infection by Lassa virus (LASV), a member of the *Arenaviridae*, causes a range of clinical outcomes from a mild or inapparent febrile illness to Lassa fever (LF), an often-fatal hemorrhagic disease. LASV is endemic in Nigeria, Sierra Leone, Guinea, and Liberia with sporadic cases reported in other West African countries^1–5^. The main reservoir of LASV is *Mastomys natalensis*, a peridomestic rodent that is abundant across a wide range of sub-Saharan Africa^6,7^. Additional rodent reservoirs or intermediate hosts have also been described^7,8^. Infection occurs by exposure to rodent urine or feces or during preparation of the rodent for food. There have been advances in understanding the structural biology, immunology and genomics of LASV^2,9,10^, progress towards an immunotherapeutic drug^11,12^ and new initiatives to develop a LF vaccine^13^. However, there is as yet no approved vaccine, and the only available treatment is off-label use of the nucleoside analog drug ribavirin^14^.

Prompt diagnosis of LASV infection is imperative for proper clinical management and isolation of patients^15^. Treatment with ribavirin, when available, must be initiated early in the disease course, and survival is improved with supportive care including replacement of fluids and electrolytes or dialysis. Early diagnosis on the basis of clinical presentation alone is challenging because the initial symptoms, including fever, headache, malaise and general weakness, are nondescript^15–19^ and common to other febrile illnesses in West Africa, such as malaria, typhoid fever, leptospirosis and arbovirus diseases^20^.

Laboratory diagnosis of acute LF in West Africa relies on the detection of LASV antigen (Ag) using immunoassays or genomic RNA using molecular diagnostics, such as polymerase chain reaction (PCR)^21–25^. We recently compared the field performance of recombinant antigen-based LF immunoassays to that of quantitative PCRs (qPCRs) using samples from acutely ill subjects meeting the case definition of LF presenting to Kenema Government Hospital (KGH) in Kenema, Sierra Leone. Zalgen Lab’s first generation ReLASV RDT is a lateral flow immunoassay, based on paired murine monoclonal antibodies (MAb) to the Josiah strain of LASV (lineage IV); it performed with 90% sensitivity and 100% specificity, better than the most robust qPCR currently available (82% sensitivity and 95% specificity)^26^.

The diagnosis of LF by molecular and immunological methods is challenged by the high genetic diversity of LASV^2,27^, which is greatest in Nigeria. Preliminary studies suggested that a Pan-Lassa RDT configured with polyclonal antibodies and designed to recognize LASV of divergent lineages, including those in Nigeria, could have improved sensitivity and specificity compared to a Mab based RDT^26^. In 2018, a dramatic increase in the number of reported LF cases occurred in Nigeria, providing an opportunity to more thoroughly test a Pan-Lassa RDT there. The Nigerian Center for Disease Control (NCDC) reported 431 cases from 21 states during the first four months of 2018^28^. The factors underlying the increase in cases are not known. Genomic analysis found no evidence that a particular LASV strain or extensive human-to-human transmission drove the increase^29,30^. LASV transmission during the 2018 surge in Nigeria was sustained largely by numerous distinct cross-species transmission events from a genetically diverse reservoir. Here, we compare the performance of the Pan-Lassa RDT designed to detect infection with the most common LASV lineage to available quantitative PCR (qPCR) assays during the 2018 LF outbreak in Nigeria.

## Results

### A cohort of suspected LF cases presenting to Irrua Specialist Teaching Hospital

We assembled a set of samples from suspected LF cases who were referred for testing to the Institute of Lassa Fever Research and Control at Irrua Specialist Teaching Hospital (ISTH) in 2018. Patient samples (N = 434) that had sufficient volume to run multiple diagnostic assays were included in the current analysis. Available patient data included age, sex, date of presentation, state of residence, estimated time to diagnosis, symptoms on presentation and outcome; the metadata was incomplete for 11% (49/434) of the patients. The set of stored samples collected during the 2018 Nigeria LF outbreak was used to evaluate the diagnostic potential of a suite of diagnostic immunoassays based on recombinant LASV proteins (ReLASV)^1,20,25,31^.

**Figure 1:**
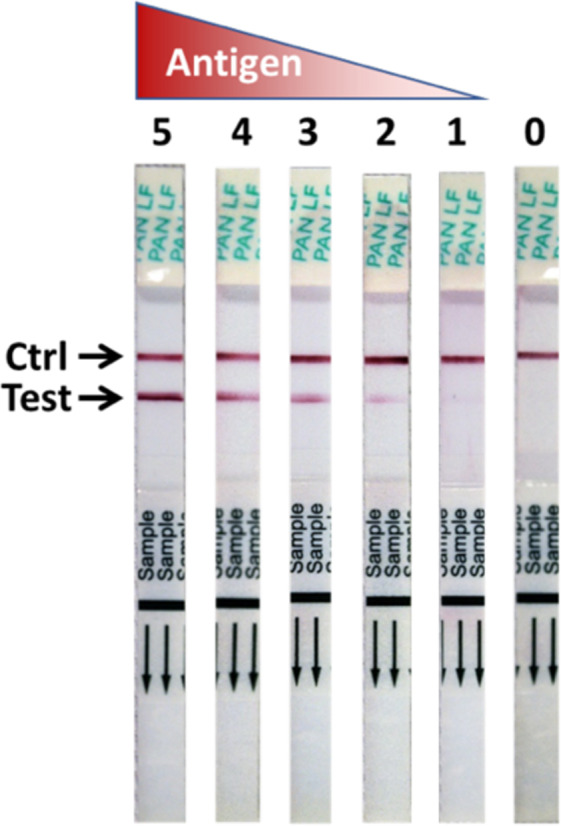
Signal development of the ReLASV Pan-Lassa antigen rapid diagnostic test. The ReLASV Pan-Lassa Antigen RDT is designed as a dipstick style lateral flow immunoassay. It can be visually scored on a scale of 0 to 5.

ReLASV IgM and IgG capture ELISAs utilize microwell plates coated with a mixture of recombinant LASV nucleoprotein (NP)^31,32^. The Pan-Lassa RDT is a dipstick-style lateral flow immunoassay using rabbit polyclonal antibodies to LASV NP of lineages II, III and IV, the most prevalent lineages in Nigeria and Sierra Leone. The Pan-Lassa RDT can be visually scored on a scale of 0 to 5, with strong positive samples developing in as little as 5 minutes and full signal development by 25 minutes (Fig. 1).

Quantitative real-time PCR (qRT-PCR) assays for LF diagnosis have been introduced by Trombley *et al*.^33^, Nikisins *et al*.^34^ and Altona 1.0^35^ These and other qRT-PCR assays are in various stages of development. During 2018, the ISTH Lassa Program screened suspected LF cases using two qRT-PCR methods, the RealStar Lassa Virus RT-PCR Kit 1.0 (aka Altona 1.0; Altona Diagnostics GmbH, Hamburg, Germany) and the RT-PCR method of Nikisins *et al*.^34^.

**Figure 2:**
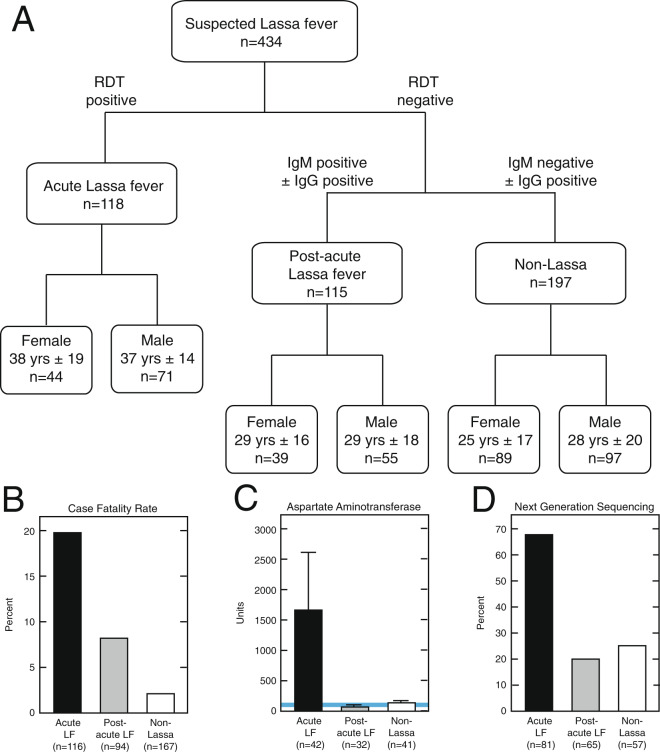
Characteristics of subjects from 2018 outbreak of Lassa fever presenting to Irrua Specialist Teaching Hospital in Nigeria. Panel A: Demographics of subject cohort classified by Lassa immunoassay results. Subjects were classified as Acute Lassa fever, Post-Acute Lassa fever, and Non-Lassa Illness based on ReLASV immunoassay screening. Panel B: Case fatality rates of subjects classified by Lassa immunoassay results. Panel C: Aspartate Aminotransferase levels in samples from subjects classified by Lassa immunoassay results. Panel D: Detection of LASV genomes (>400 reads) by Next Generation Sequencing from samples of subjects classified by Lassa immunoassay results.

For the current analysis, Pan-Lassa RDT, Pan-Lassa antibody-capture immunoassay data and qPCR data were analyzed from the cohort of 434 subjects (Fig. 2). Four samples were excluded from further analysis because of discordant qPCR results. The remaining sample set (n = 430) was subdivided based on the results of the ReLASV screening immunoassays (Fig. 2A). Patients that were positive on the Pan-Lassa RDT were classified as Acute LF (n = 118, Table S1). Patients were classified as Post-acute LF where RDT were negative, but anti-LASV IgM antibodies where positive (N = 115, Table S2). The Non-LF illness group includes patients that were negative on all ReLASV immunoassays and patients with only anti-LASV IgG antibodies (N = 197, Table S3). There were more males than females in each group (Fig. 2A). Patients with Acute LF were also older than patients in other groups, but a broad distribution of ages was represented in each group. The differences between groups by sex and age were not significant. The average interval from symptom onset to diagnosis of patients with acute LF was 7.5 ± .35 days (standard error of the mean). This interval was significantly greater (p < .0001) than the 5.6 ± 0.30 day interval for subjects presenting with non-LF illness, but similar to the 7.5 ± 0.56 day interval for subjects with post-acute LF. There was no significant correlation between day of onset and either RDT score or level of IgM production.

The case fatality rate (CFR) in the Acute LF group was 19.8% (23/116), which was higher than in the Post-acute LF 8.5% (8/94) and Non-LF groups 2.4% (4/167) (Fig. 2B). In addition to the immunoassays and qPCR assays, results of a clinical chemistry panel were available for a subset of samples (n = 117). Previous case studies have suggested that elevated liver transaminases and kidney creatinine are markers for Acute LF^25,36^. Aspartate aminotransferase (AST) levels (mean ± SE) were significantly higher in samples from patients in the Acute LF group (1625 ± 965 U/L) than in samples from the Post-acute LF (62 ± 8 U/L) and Non-LF groups (139 ± 22 U/L) (Fig. 2C). A subset of samples was also subjected to next generation sequencing analyses (NGS)^29^. Samples from the Acute LF group in which NGS was attempted produced 55 (68%) LASV genomic sequences, versus 13 (20%) and 15 (26%) of the Post-acute LF and Non-LF groups, respectively.

**Figure 3:**
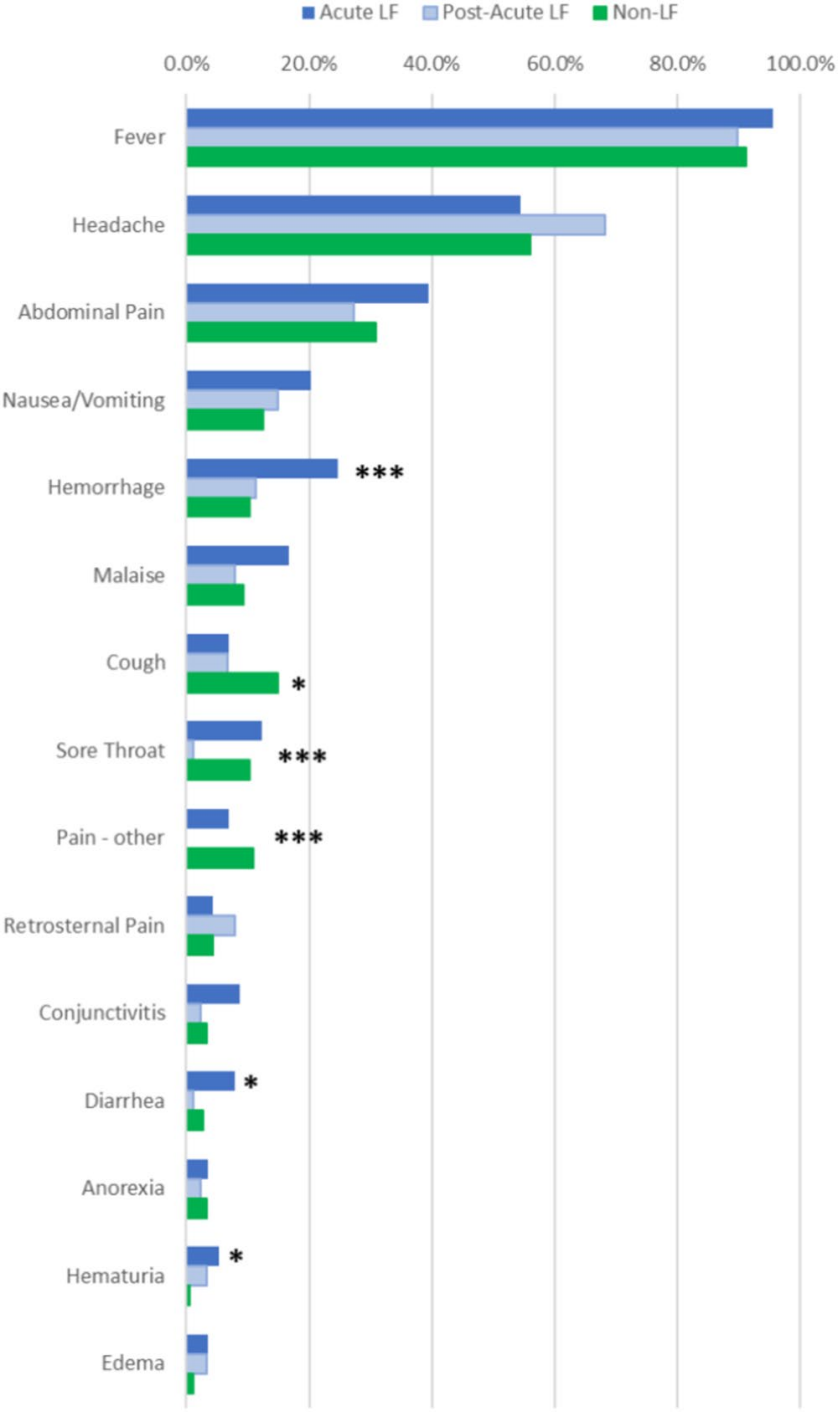
Reported clinical signs and symptoms in the 2018 cohort of suspected Lassa fever cases presenting to Irrua Specialist Teaching Hospital. Subjects were classified as Acute Lassa fever, Post-Acute Lassa fever, and Other Illness based on Lassa immunoassay screening. Asterisks represent P > ChiSq for difference between three patient groups (*p < 0.05; ***p < 0.001).

Fever was the most common symptom reported in each group in the cohort and it was present in over 90% of patients (Fig. 3). The next most common symptom was headache, which was present in over half of all patients. Abdominal pain was present in approximately one-third of subjects in each group. No other symptom, except hemorrhage, was present in more than one-fourth of the patients of any cohort. Hemorrhage was present in 25% of Acute LF patients. Although this difference was significant (p < 0.0001) when compared to the Post-acute LF (10%) and the Non-LF (11%) groups, the low frequency of hemorrhage limits its utility for differential diagnosis (Table S4). Additional symptoms with significant differences between the Acute LF, Post-acute LF and Non-LF groups, including cough, sore throat and pain, but these symptoms were also present in low frequency providing weak LF screening utility.

### Comparison of LASV RT-qPCR to genomic sequencing results

**Figure 4:**
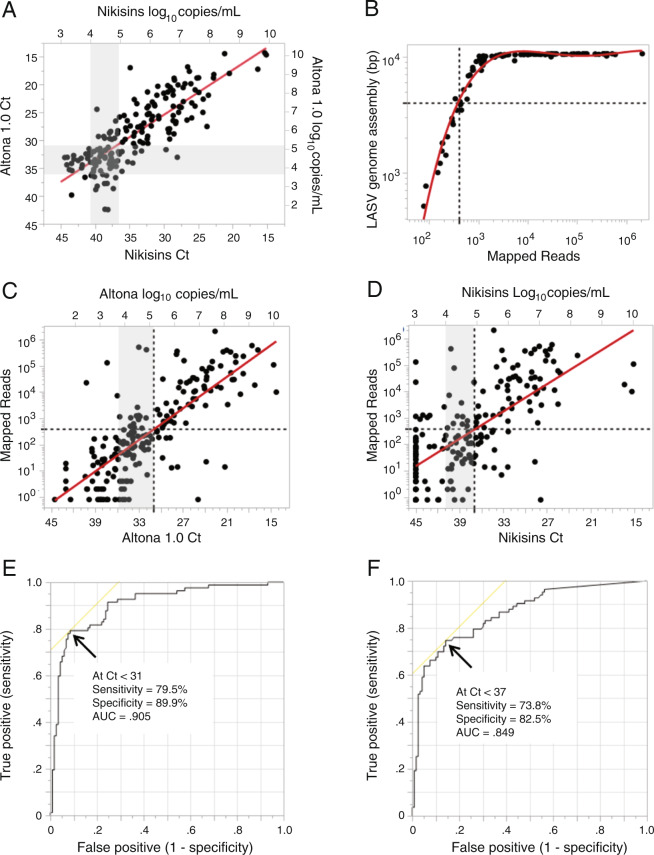
Correlations of quantitative polymerase chain reaction assay results with Next Generation sequencing of Lassa virus genomes. Panel A: Correlation between Altona 1.0 qPCR and Nikisins qPCR cycle threshold (Ct) results and log_10_ of viral genome equivalents per mL (n = 178, R^2^ = 0.64, Linear Regression = 1.80 + 0.79). Panel B: LASV genome assembly length correlation to post-filtering mapped reads (cubic fit of Log-Log transformation of data, R^2^ = 0.97). Panel C: Log – Linear transformed linear fit of Altona 1.0 qPCR Ct and log_10_ of viral genome equivalents per mL to mapped reads (n = 199, R^2^ = 0.60). Panel D: Log – Linear transformed linear fit of Nikisins qPCR Ct and log_10_ of viral genome equivalents per mL to mapped reads (n = 201, R^2^ = 0.45). Panel E: Receiver operator curve determination of Altona 1.0 qPCR Ct cut-off based on LASV genomic sequencing. Panel F: Receiver operator curve determination of Nikisins qPCR Ct cut-off based on LASV genomic sequencing.

Any positive fluorescence value obtained below a 43-cycle threshold (Ct < 43) for the Altona 1.0 qPCR, or a 45-cycle threshold (Ct < 45) for the Nikisins qPCR, was considered a positive result in the initial round of patient screening at ISTH. We compared the two qPCR assays with each other and with the results of next generation sequencing (NGS) (Fig. 4). Correlation of the two qPCR methods was good for paired Ct’s (excluding non-reactive samples) with R^2^ = 0.639 with linear regression analysis showing a +21% bias in Ct value for the Nikisins method (Fig. 4A).

Although not used in the initial screen, a cutoff value for numbers of cycles (Ct) is traditionally utilized in qPCR assays. In a concurrent metagenomic sequencing study, ISTH qPCR positive samples were submitted for NGS at African Center of Excellence for Genomics of Infectious Diseases (ACEGID), Redeemers University, Nigeria and Broad Institute/Harvard University, USA^29^. For the NGS analysis, an assembly length equivalent to ≥4000 base pairs, which corresponded to ≥400 reads mapped to the template genome, was used as a benchmark for successful genome identification (Fig. 4B). Use of ≥400 mapped reads correlated to qPCR Ct cut-offs of Ct ≤31 for Altona 1.0 or approximately 10^5^ viral genome copies/mL (Fig. 4C) and Ct ≤ 37 for Nikisins qPCR, which is also approximately 10^5^ viral genome copies/mL (Fig. 4D), was determined using logistic regression and receiver operator characteristic (ROC) curve analysis (Fig. 4E,F). These cutoffs are consistent with previously determined limits of detection of Lassa qPCR assays^37,38^. Contingency analysis of the proposed qPCR cut-off at the optimal diagnostic likelihood (sensitivity/1-specificity) compared to NGS reveals sensitivity = 79.5% and specificity = 89.9% for Altona at Ct ≤ 31 (Fig. 4E, Table S5). Performance of Nikisins qPCR at Ct ≤ 37 is sensitivity = 73.8% and specificity = 82.5% (Fig. 4F, Table S6). There was a good correlation between the number of reads that mapped to the LASV genome and the number of mapped reads per million (RPM) total reads (Fig. S1). Similar cutoffs for both qPCRs were obtained using either total number of reads or RPM.

An equivocal (indeterminate) range greater than the Ct cutoff may also be employed for qPCR assays^39,40^. An equivocal range for the two qPCR methods was based on the Ct distribution of samples that were positive by the assigned cut-off of the competing qPCR method. Thus, the Altona Ct distribution for samples positive by Nikisins qPCR estimates an upper limit of the equivocal range as Altona Ct = 36 (Fig. S2). Similarly, the Nikisins Ct distribution for Altona 1.0 positive samples estimates an upper limit of the equivocal range as Nikisins CT = 41 (Fig. S3).

**Figure 5:**
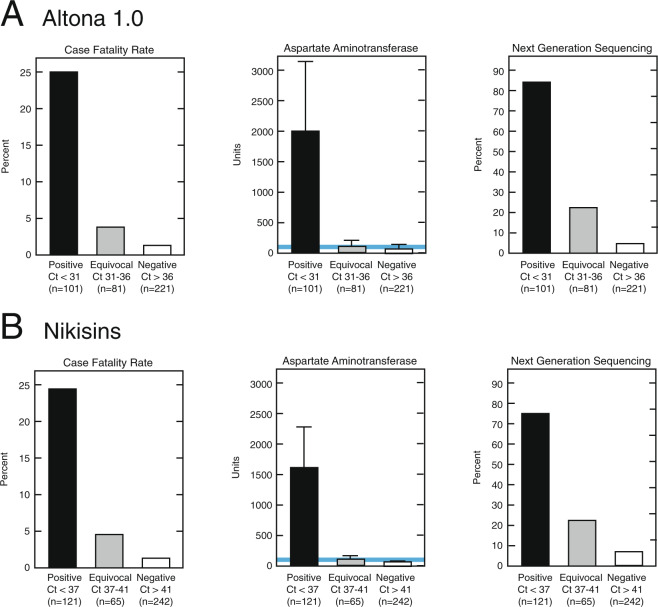
Demographics of the 2018 cohort of suspected Lassa fever cases presenting to Irrua Specialist Teaching Hospital classified by Lassa quantitative polymerase chain reaction results. Samples from subjects were classified as positive, equivocal or negative on the Altona 1.0 qPCR (Panel A) or the Nikisons qPCR (Panel B). Case fatality rates of subjects by qPCR results are compared. Aspartate aminotransferase levels and presence of LASV sequences in samples from subjects are also compared.

Patients whose samples were positive on the qPCR assays differed from patients whose samples were equivocal or negative by CFR, liver transaminases and NGS (Fig. 5). The CFR for suspected LF patients whose samples were positive on the Altona 1.0 qPCR assay was 25% (25/100), which was higher than in the equivocal samples with 4% (3/79) and negative samples with 2% (3/172) (Fig. 5A). AST levels were significantly higher in samples from patients whose samples were positive (mean = 1911 U/L) on the Altona 1.0 qPCR, than in equivocal (mean = 97 U/L; p = 0.0490) or negative samples (mean = 63 U/L; p = 0.0466). LASV genomic sequences were produced in 84% (65/77) of Altona 1.0 positive samples by NGS, compared to 21.1% (15/71) of equivocal samples and 4% (2/52) of negative samples. Similar results were obtained for samples that were positive, equivocal and negative by the Nikisins qPCR (Fig. 5B).

### Comparison of Pan-Lassa RDT with qPCR

**Figure 6:**
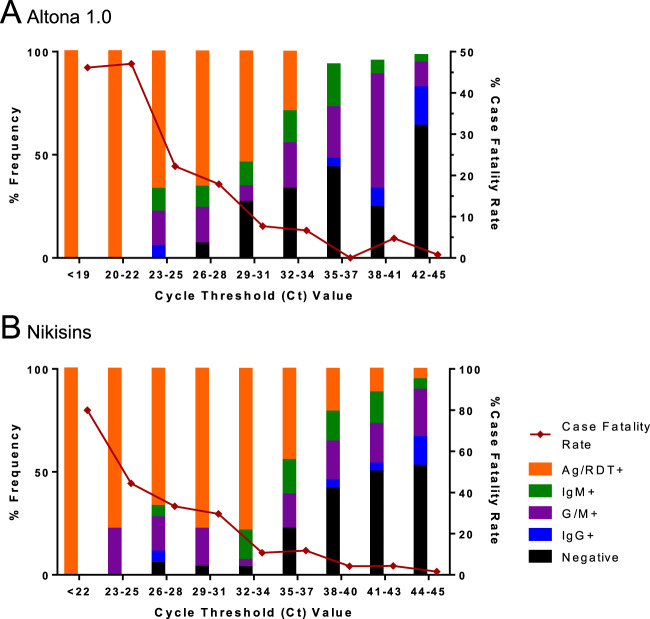
Relationship of Pan-Lassa rapid diagnostic test and antibody capture immunoassay results to quantitative polymerase chain assay results. Samples from subjects presenting to Irrua Specialist Teaching Hospital with suspected Lassa fever in 2018 were used to compare results of the recombinant Lassa immunoassays to results of the Altona 1.0 qPCR assay (Panel A) and the Nikisins qPCR assay (Panel B).

Across the entire dataset there was a strong relationship between low Ct values, which reflect high viral RNA levels, and positivity on the Pan-Lassa RDT. All samples with a Ct value below 22 on both the Altona 1.0 and Nikisins qPCR were positive on the Pan-Lassa RDT (Fig. 6A,B). In contrast, only 10% of samples with an Altona qPCR Ct above 35 were positive on the Pan-Lassa RDT. Similar results were obtained with the Nikisins qPCR above 43. The Ct range covering the transition from LASV antigenemia to IgG/IgM seroconversion spans approximately Ct 23–37 for Altona 1.0, but Ct 26–43 for Nikisins qPCR. Ct values for all samples are shown in Tables S1, S2, S3.

This transition from LASV antigenemia during acute viremia to IgM and IgG seroconversion is reflected by the inverse correlation of the RDT signal vs IgM and IgG titers (Fig. S4). There was also a strong inverse relationship between low Ct values (high viral RNA) and the presence of anti-LASV IgM or IgG (Fig. 6A,B). None of the samples with a Ct value below 22 and only a third of the samples with Ct values of 23–28, tested positive for LASV-specific IgM or IgG. Conversely, more than half of the samples with Ct values above 35 by either qPCR were positive for LASV-specific IgM or IgG; or were immunoassay naive. These results indicate that the presence of a humoral immune response to LASV clears viral antigen measured by the Pan-Lassa RDT and signals potential reduction of circulating viral RNA, as measured by the qPCRs, due to neutralization of viremia. A significant correlation between day of symptom onset and results on either qPCR assay or the RDT was not observed in any of the three cohorts (acute LF, post-acute LF or non-LF).

There was a strong relationship between qPCR Ct values, RDT positive rates and CFR. For patients presenting with Altona 1.0 Ct values below 19, all of which were Pan-Lassa RDT positive, the CFR was 45% (Fig. 6A). The CFR dropped below 8% in cases with a CT above 35 and to less than 1% in case whose samples were negative on both the Altona qPCR (Ct 43–45) and the Pan-Lassa RDT. A similar relationship was observed for the Nikisins qPCR (Fig. 6B). These results are consistent with prior studies which have shown there is a strong correlation between presentation with high LASV load and the risk of death from LF, as well as a strong correlation with the presence of a humoral immune response and probability of survival^1,24^. These results also confirm the clinical utility of both the qPCR and RDT assays and suggests that the relative performance of the two types of assays are comparable.

### Clinical performance of Pan-Lassa RDT

**Figure 7:**
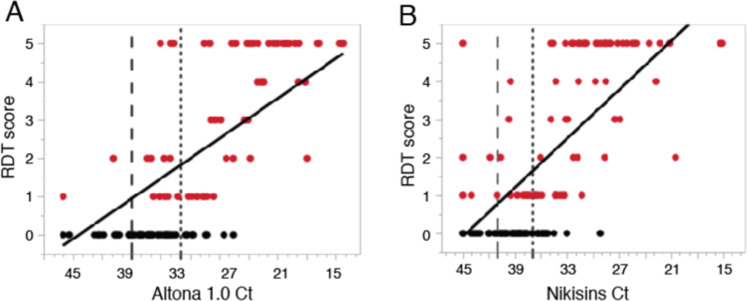
Correlation of quantitative polymerase chain reaction cycle threshold values and Pan-Lassa rapid diagnostic test signal intensity. Signal intensity based on a visual score aid for the Pan-Lassa RDT was compared to cycle threshold (Ct) values for the Altona 1.0 (R^2^ = 0.65; Panel A) and Nikisins qPCR (R^2^ = 0.62; Panel B) for samples from subjects with acute Lassa fever (IgG seronegative).

The Pan-Lassa RDT was developed to address the unmet need for a rapid point-of care test to triage potentially acute cases of LF caused by genetically divergent LASV across the broad LF range in West Africa. Since a large cohort of the study group is in a post-acute stage of LF, the most accurate performance estimate is determined by comparing the RDT to the qPCR reference method for samples that are IgG and IgM seronegative (Fig. 7). A substantial linear correlation was observed between the Ct values of the Altona qPCR assay and visual scoring of the Pan-Lassa RDT (R^2^ = 0.653, Fig. 7A) for IgM/IgG seronegative samples. A similar degree of correlation was observed between the Nikisins qPCR assay and the Pan-Lassa RDT (R^2^ = 0.620, Fig. 7B).

Performance estimates of the Pan-Lassa RDT were determined using the Altona 1.0 and Nikisins qPCR as separate reference methods with and without Ct cut-offs previously established (Table 1). RDT performance was also compared to composite qPCR results to account for samples that are equivocal by one qPCR but positive by the alternate qPCR method. The clinical performance was estimated for IgG/IgM seronegative samples to assess utility in screening acute LF cases. Limiting the performance estimates to IgG/IgM seronegative samples to assess triage of acute LF improves overall diagnostic performance. Compared to Nikisins qPCR, the Pan-Lassa RDT is 84.5% sensitive and 89.3% specific; compared to Altona 1.0 qPCR the Pan-Lassa RDT is 85.3% sensitive and 85.6% specific. The Pan-Lassa RDT performance, when compared to the combined qPCR results of Altona and Nikisins methods, for IgG/IgM seronegative samples is sensitivity of 83.3% and specificity at 92.8%, with a diagnostic likelihood ratio of 11.6.

**Table 1:** Performance of Lassa fever diagnostic assays using different diagnostic standards.

ReLASV Antigen RDT Performance	With Ct Cut-off and Equivocal Range	With Ct Cut-off (G/M sero-negative)
**Nikisins qPCR**		
Sensitivity	69.1% (60.1–77.1%)	84.5% (74.0 – 92.0%)
Specificity	88.8% (84.7 – 92.0%)	89.3% (83.8 – 93.4%)
PPV	70.8% (61.8 – 78.8%)	76.0% (65.0 – 84.9%)
NPV	87.9% (83.8 – 91.3%)	93.5% (88.7 – 96.7%)
Dx Likelihood	6.14	7.87
%G/M+	42.3%	0%
**Altona qPCR**		
Sensitivity	72.8% (63.2 – 81.1%)	85.3% (73.8 – 93.0%)
Specificity	86.4% (82.2 – 89.9%)	85.6% (79.7 – 90.3%)
PPV	62.5% (53.2 – 71.2%)	65.8% (54.3 – 76.1%)
NPV	91.1% (87.4 – 94.0%)	94.7% (90.1 – 97.5%)
Dx Likelihood	5.36	5.90
%G/M+	40.8%	0%
**Composite qPCR**		
Sensitivity	70.2% (61.6 – 77.9%)	83.3% (73.2 – 90.8%)
Specificity	91.3% (87.5 – 94.2%)	92.8% (87.8 – 96.2%)
PPV	78.0% (69.4 – 85.1%)	84.4% (74.4 – 91.7%)
NPV	87.5% (83.3 – 91.0%)	92.3% (87.1 – 95.8%)
Dx Likelihoo	8.08	11.6
%G/M+	40.5%	0%
**LASV qPCR Performance vs RDT**	**Altona qPCR**	**Nikisins qPCR**
**RDT as Ref. Method**	Ct ≤ 31	Ct ≤ 37
Sensitivity	76.9% (64.8 – 86.5%)	77.9% (67.0 – 86.6%)
Specificity	94.4% (89.7 – 97.4%)	94.0% (89.3 – 97.1%)
PPV	84.8% (73.0 – 92.8%)	85.7% (75.3 – 92.9%)
NPV	91.0% (85.6 – 94.9%)	90.2% (84.8 – 94.2%)
Dx Likelihood	13.76	13.01
%G/M+	0%	0%

*Ct cut-off based on ROC comparison of NGS and qPCR.

## Discussion

This study evaluated a suite of LF immunoassays based on LASV recombinant proteins using samples from patients referred to the Lassa surveillance program at ISTH during the 2018 LF outbreak in Nigeria^1,20,25,31^. Following screening of the suspected LF cases by qPCR, stored samples were tested on the Pan-Lassa RDT, which utilizes a mixture of polyclonal antibodies raised against the nucleoproteins of representative strains from the three most prevalent LASV lineages (II, III and IV). An earlier generation lateral flow immunoassay, based on paired monoclonal antibodies to the Josiah strain of LASV (lineage IV), performed with high sensitivity and specificity in diagnosing cases of LF presenting to KGH in Sierra Leone^26^. However, this lineage IV specific RDT performed with reduced sensitivity for diagnosis of suspected LF patients in central Nigeria, which is predominantly LASV lineage II. Further studies are needed to define the sensitivity of the Pan-Lassa RDT on lineage I and lineage III LASV isolates and newly described LASV lineages V-VII^7,41,42^. For patients with acute LF, the Pan-Lassa RDT performance was 83.3% sensitivity and 92.8% specificity when compared to composite results of two qPCR assays. 100% of samples that gave Ct values below 22 on both qPCR assays were positive on the Pan-Lassa RDT.

This current study confirms that LF is difficult to diagnose on the basis of clinical signs and symptoms^1^. Moreover, reliance on central laboratory testing can delay diagnosis of LF patients by hours or days, making a point-of-care RDT even more valuable. The field performance of the Pan-Lassa RDT suggests that it may be a useful tool for triage of suspected LF cases and management of patients with LF. Diagnosis using the Pan-Lassa RDT is not dependent on sample processing, such as extraction of nucleic acids, or instrumentation that requires a stable source of electrical power, as is the case with PCR.

There are a number of limitations to the current study. While each patient was referred and enrolled for LASV testing during an outbreak situation, fewer than half (37%, 143/385) of the subjects in the cohort that were evaluated met the case definition of suspected LF upon inspection of the metadata. Supporting laboratory data, such as clinical chemistries, was available from only a subset of patients. Approximately half (48%, 184/386) of patients for which limited demographic and clinical data was available were patients who were not admitted to the Lassa Ward. This was a cross-sectional study and only one sample was tested per patient. Future validation studies of Pan-Lassa diagnostic assays should include longitudinal samples collected later in the disease to confirm the presence or absence of LASV infection.

The Pan-Lassa RDT has potential utility in settings where nosocomial infections from LF patients pose an immediate threat to health care workers and in outbreak settings. The Pan-Lassa RDT detects patients with a high virus load with a high sensitivity and specificity. Patients with elevated levels of LASV present the highest risk for transmitting LASV to health care workers (HCW). Pregnant women often have high virus load in blood and other bodily fluids, and are at increased risk for developing severe LF^1,43,44^. The use of RDTs in maternity wards in LF endemic regions could aid in identification of potential cases and limit HCW transmission. The Pan-Lassa RDT may also find utility in other clinical settings where exposure to bodily fluids may occur, such as surgical units. Patients who test positive for LF by RDT should be transported to medical facilities where confirmatory tests and enhanced supportive care and infection control measures are available. Use of the Pan-Lassa RDT can provide a presumptive diagnosis that could lead to earlier treatment with the antiviral drug ribavirin^1,45^ or LF drugs in under development^11,12,46^. LASV-specific IgM or IgG immunoassays can also contribute to clinical management of LF patients. Post-acute LF patients are particularly challenging to manage as their presentation can include detectable levels of viral nucleic acid, circulating antigen, IgM, IgG, or a combination thereof. Patients at the post-acute stage of LASV infection whose immune response has controlled the LASV load, yet remain sick enough to seek treatment, may not benefit from ribavirin treatment. These later stage patients may, however, benefit from supportive therapies such as fluid replacement.

A proposed diagnostic algorithm follows from the use of the Pan-Lassa RDT and ELISAs. Under this scenario, suspected LF cases would be triaged using the Pan-Lassa RDT. Presumptive results of the RDT would be confirmed by qPCR using pre-established Ct cut-offs along with concurrent ELISA screening to designate the patients as Acute LF, Post-Acute LF, or Non-LF (including LF Convalescent, IgG+). Additionally, if available, serum chemistry panels can help support a diagnosis of acute LF and help guide supportive care measures. This screening algorithm has the potential to reduce High Containment LF Ward case load compared to the current approach, which designates any qPCR reactivity as a confirmed LF case. These decisions can begin to be made in the development time of the Pan-Lassa RDT, as little as 5–25 minute.

The current study also informs the use of qPCR based assays for LF diagnosis. The results support both the use of a Ct cutoff and equivocal range for the Lassa qPCRs, as has been employed for qPCRs assays for other viral hemorrhagic fevers such as Ebola^39,40^. A unique feature of the current study was the availability of concurrent NGS data from a subset of the cohort. Most of the samples tested in the current cohort exhibited a qPCR signal on either the Altona 1.0 or Nikisins qPCR. However, samples giving Ct values above our calculated cut-offs or samples with equivocal qPCR results exhibited substantially reduced frequency of successfully detecting LASV genomes during NGS compared to qPCR positives. This suggests that a subset of these samples do not contain LASV nucleic acids. Off-target amplification, generation of primer dimers and other artifacts may produce low-level signals in qPCR, often characterized by non-logarithmic amplification. In a recent study it was determined that samples that produced Ct values above the cut-offs established here did not contain replication-competent LASV (in preparation). Further studies to optimize and evaluate Lassa qPCR assays for divergent lineages of LASV, including the use of assays that combine more than one set of PCR primers, would help reduce the ambiguity of results in the negative or equivocal ranges. Development of more sensitive methods for detection of LASV genomic RNA should also proceed with an emphasis on platforms that are low cost, and can be deployed and maintained in austere environments.

The imperfect performance of the qPCR, coupled with the increasing genetic diversity of LASV across West Africa, highlights the challenges of evaluating the performance of Lassa diagnostics^47–49^. A recent sequencing study of LASV genomes from Liberian LF patients identified concerns in the current molecular diagnostic assays being used in Liberia^50^. In particular, when compared to primers used in various qPCR assays, numerous nucleotide mismatches present in recently circulating LASV genomes were identified that may impact amplification. The current results support the call to evaluate more recently developed pan-Lassa qPCR assays and immunological tests in Liberia, and to develop a more comprehensive testing algorithm.

Neither qPCR based assays nor NGS represent ideal diagnostic standards to appropriately evaluate the sensitivity and specificity of either LASV immunoassays or nucleic acid tests. In that regard, the estimate of Pan-Lassa RDT performance (83.3% sensitivity and 92.8% specificity) should be considered in the context that the PCR assays represent an imperfect standard. While PCR-based assays are often assumed to be more sensitive than immunoassays, the latter can be far more sensitive in the post-acute stage of the disease. This situation was evident in a previous study of samples from a recent cohort of Sierra Leonean patients with suspected LF^26^. Many of the subjects in the current cohort were in the post-acute stage of disease. Persistence of LASV antigen, genomic RNA and anti-LASV IgM may occur in a subset of subjects even after clearance of infectious virus, has been observed in other virus infections^40,51,52^. For example, a recent report showed that it was possible to isolate replicating Ebola virus from samples with a Ct ≤ 30, but not from samples with a Ct> 33 with a grey zone with sporadic isolations from samples with a Ct 30–32^40^. In a subset of samples from subjects examined here, LASV nucleic acids or antigen were detected for extended periods into the convalescence stage. LASV infections have been characterized when circulating antigenemia precedes detection of viral nucleic acid, but in most instances the reverse pattern is commonplace. Additionally, nucleic acid is commonly detected after serum antigenemia subsides, although in a significant number of cases shed viral antigen can be detected after clearance of nucleic acid and infectious virus^26^.

Development of the Pan-Lassa RDT and ELISAs with polyclonal antibodies results in sensitive and specific immunodiagnostic platforms that are more tolerant to genetic variation than PCR assays. The expected overall higher sensitivity of PCR-based assays can extend the detection range of LASV infections, particularly in early (asymptomatic) and post-acute stages of LF. Therefore, both platforms should be part of a comprehensive diagnostic algorithm in the testing of suspected LF patients. A combined testing algorithm has been previously proposed toward a best available diagnostic evaluation, treatment options, and patient outcome^23^. In patients where IgM and/or IgG titers have emerged, ribavirin treatment may not result in enhanced survival^1^. This subset of patients may benefit primarily from supportive therapy.

Future studies should evaluate LF diagnostics throughout the course of infection. When possible, retesting of patients should be performed 24–48 hours after a LF diagnosis is made, with equivocal results carefully evaluated (e.g. one positive and one negative PCR, with positive or negative antigenemia, and with positive or negative IgM and/or IgM titers). Retesting these samples 24–48 hours after the initial diagnosis often clarifies the patient’s LF status. Treatment options for patients in such instances is always at the discretion of the attending physician and nursing staff.

The increasing frequency of viral hemorrhagic fever (VHF) outbreaks across sub-Saharan Africa has shined a spotlight on the need to complete the armamentarium of medical countermeasures for Lassa, Ebola and other VHFs. International groups, such as CEPI, are accelerating efforts to develop therapeutics and vaccines for LF^53^. ReLASV Pan-Lassa *in vitro* diagnostics are uniquely positioned to inform and support these efforts as a suite of robust, accurate medical devices to further expand LF surveillance throughout LF-affected West African countries. Our findings demonstrate the clinical utility of the Pan-Lassa RDT as well as the Pan-Lassa NP IgG/IgM ELISAs. Future studies to validate these assays to aid in diagnosis of LF, case investigations, and epidemiology studies can contribute to improved LF disease management and control in conjunction with vaccine and therapeutic candidates under development.

## Methods

### Study design

Human subjects research was conducted in accordance with all relevant guidelines and regulations, including the Declaration of Helsinki. Clinical research including all human subjects testing was approved by ISTH, Redeemer’s University, Harvard University and the Tulane University Institutional Review Boards (IRB). All patients enrolled in this study and/or their legal guardians provided written informed consent after the nature and possible consequences of the studies were explained. Excess clinical samples (deidentified, surplus diagnostic samples) were obtained under a waiver of consent granted by the ISTH Research Ethics Committee. All samples were deidentified prior to qRT-PCR screening and performance of the immunoassays in the ISTH Lassa fever laboratory that operates at Biosafety level 2 plus (BSL-2+). Demographic, clinical, and laboratory data were obtained in accordance with ethics approval. Samples were obtained prior to administration of ribavirin, which reduces virus load in some patients. Only ISTH staff were involved in the administration of health care to suspected Lassa fever patients at the ISTH Lassa Ward. All medical decisions, including whether or not to administer ribavirin to patients, were at the sole discretion of the attending ISTH Lassa Ward physicians.

### ReLASV RDT

The ReLASV Pan-Lassa Antigen Rapid Test (RDT) has been developed using affinity purified polyclonal rabbit antibodies specific for LASV nucleoprotein (NP) antigen^25,31^. The immunochromatographic dipstick design incorporates a plasma separator sample pad, a gold nanoparticle-labelled PAb, a test line consisting of a PAb that captures the LASV NP antigen-PAb nanoparticle complex, and a rabbit IgG specific control line. 30 µL of whole blood, plasma, or serum is introduced onto the sample pad, and the dipstick is then inserted into a culture tube containing 200 µL (4 drops) of sample buffer, which initiates the flow of sample and sample buffer. Incubation time is 15–25 minutes at ambient temperature (18–30 °C) for full signal development. Results are scored on a scale of 0–5 using a visual aid (Fig. 1). In future studies we will evaluate the utility of a mobile phone application ‘HandLens’ that captures and analyzes an image of one or more lateral flow strips to quantify test results, facilitate accurate readout and resolve ambiguous readouts^54^.

### ReLASV IgM and IgG ELISA

The ReLASV Pan-Lassa NP-specific IgM and IgG ELISA utilizes microwell plates coated with a mixture of recombinant NP lineage II, III, and IV^24^. The calibrators, controls and patient serum are diluted 1:100 in sample buffer. Diluted calibrator, controls, and samples are transferred into the microwell plate (100 µL/well) and incubated for 30 minutes at ambient temperature (18–30 °C). Microwells are washed four times with 300 µL/well of PBS-Tween wash solution. Peroxidase labeled human IgG or IgM Fc-specific caprine polyclonal reagent (Jackson ImmunoResearch Laboratories, Inc. West Grove, PA) is added to the microwells (100 µL/well) and incubated at ambient temperature for 30 minutes. Microwell wash step is repeated. Soluble TMB substrate is added (100 uL/well) and incubated 10 minutes followed by addition of Stop Solution (100 uL/well). Microplates are read at 450 nm with 650 nm subtraction. IgG and IgM concentration (Units/mL) are estimated using a 4-parameter logistic fit. Negative cut-offs based on normal controls are equal to IgG >6.5 U/mL and IgM >5.6 U/mL.

### LASV quantitative polymerase chain reaction assays

LASV quantitative PCR at ISTH was previously described in Kafetzopoulou *et al*., 2019^30^. Briefly, two independent qPCR methods are used to evaluate the presence of Lassa viral genomes in the ISTH Lassa fever laboratory that operates at Biosafety level 2 plus (BSL-2+). The Altona 1.0 qPCR assay (RealStar Lassa Virus RT-PCR Kit 1.0 CE, Altona Diagnostics, Hamburg, Germany) targets the LASV S gene segment with non-degenerate primers and probe. The Nikisins qPCR assay targets the L gene segment and utilizes a set of highly degenerate primers and probe^34^. Viral RNA was extracted using QIAamp Viral RNA Mini Kit according to manufacturer’s instructions (Qiagen). Nikisins qPCR is optimized to use the Invitrogen SuperScript III Platinum One-Step qRT-PCR reagents (ThermoFisher Scientific, Waltham, Mass. USA). Temperature profile for both assays followed Altona 1.0 protocol using the Rotor-Gene Q PCR cycler (Qiagen N.V., Hilden, Germany). Log_10_ genome copies per mL derived from Ct values were obtained by comparison to samples with known gene copy numbers.

### Next generation sequencing

LASV genomic sequencing methods for this study were previously described in Siddle *et al*., 2018^29^. Briefly, all clinical samples (plasma) were inactivated in buffer AVL prior to transport to sequencing labs that operate at BSL-2. Viral RNA was extracted using the QiAmp viral RNA mini kit (Qiagen) or Pathogen RNA/DNA kit (MagMax) according to manufacturer’s instructions. Contaminating DNA was removed, cDNA synthesized, and sequencing libraries prepared using the Nextera XT kit (Illumina) as previously described in Matranga *et al*., 2016^55^. Samples were sequenced using Illumina MiSeq, HiSeq. 2500, or NovaSeq machines with 100 nucleotide paired-end reads. Samples that did not successfully produce LASV genome on first attempt were not repeated. After removing reads mapping to the human genome, reads were filtered against previously known LASV genomes. Finally, de novo assembly was performed using Trinity software^56^ and scaffolded contigs against one of three LASV reference genomes representing LASV lineages II, III, IV (GenBank KM821997–8, GU481072–3, KM821772–3 respectively).

### Clinical chemistry

Blood chemistry testing was performed with a SPOTCHEM EZ analyzer (Model SP-4430; ARKRAY Inc., Kyoto, Japan) and SPOTCHEM II test strips including Liver Panel 1, according to the manufacturer’s recommendations. We performed metabolic measurements that included the blood levels of glucose, total cholesterol, amylase, glucose, blood urea nitrogen (BUN), creatinine (CRE), lactic acid dehydrogenase (LDH), alanine aminotransferase (ALT/GPT), aspartate aminotransferase (AST/GOT), albumin (ALB), total bilirubin (TBIL), and total protein (TP).

### Data analysis and statistical methods

Data analysis and statistic methods have been described previously^26^. Laboratory data, including absorbance values, were analyzed in their individual forms and were not transformed. Pairwise comparisons involving continuous variables were carried out on the ranks of the data values to account for any departures in normality or differences in standard deviations among comparison groups. Contingency analyses were performed with Fisher’s exact test. Logistic fit with Receiver Operator Characteristic (ROC) curve analysis was used to assess the diagnostic accuracy of the RDT. Data were analyzed using the JMP software (version 13.0.0, SAS Institute, Inc., Cary, NC) and Prism (version 6.07, GraphPad Software, Inc., San Diego, CA). Analyses were two-tailed with a significance threshold set at p < 0.05.

### Lassa virus strains

Representative strains from lineages II (LASV237-Nigeria-2010H), III (LASV-Nig08-A18-Nigeria-2008H), and IV (Josiah) were chosen for molecular cloning and expression of the respective nucleoprotein (NP) genes in *E. coli*. The lineage II and III strains were chosen based on their approximate median divergence from the corresponding lineage root.

## Supplementary information

Supplementary Information.

## References

[1] Shaffer JG (2014). Lassa fever in post-conflict Sierra Leone. PLoS neglected tropical Dis..

[2] Andersen KG (2015). Clinical sequencing uncovers origins and evolution of Lassa virus. Cell.

[3] WHO. Lassa Fever – Benin, Togo and Burkina Faso. Disease outbreak news 10 March 2017 (2017).

[4] Manning JT, Forrester N, Paessler S (2015). Lassa virus isolates from Mali and the Ivory Coast represent an emerging fifth lineage. Front. microbiology.

[5] ECDC. Lassa fever in Nigeria, Benin, Togo, Germany and USA. European Centre for Disease Prevention and Control 23 March 2016 (2016).

[6] Lecompte E (2006). Mastomys natalensis and Lassa fever, West Africa. Emerg. Infect. Dis..

[7] Olayemi A (2016). New hosts of the Lassa virus. Sci. Rep..

[8] Yadouleton Anges, Agolinou Achaz, Kourouma Fodé, Saizonou Raoul, Pahlmann Meike, Bedié Sonia Kossou, Bankolé Honoré, Becker-Ziaja Beate, Gbaguidi Fernand, Thielebein Anke, Magassouba N’Faly, Duraffour Sophie, Baptiste Jean-Pierre, Günther Stephan, Fichet-Calvet Elisabeth (2019). Lassa Virus in Pygmy Mice, Benin, 2016–2017. Emerging Infectious Diseases.

[9] Hastie KM (2017). Structural basis for antibody-mediated neutralization of Lassa virus. Science.

[10] Robinson JE (2016). Most neutralizing human monoclonal antibodies target novel epitopes requiring both Lassa virus glycoprotein subunits. Nat. Commun..

[11] Cross RW (2016). Treatment of Lassa virus infection in outbred guinea pigs with first-in-class human monoclonal antibodies. Antivir. Res..

[12] Mire CE (2017). Human-monoclonal-antibody therapy protects nonhuman primates against advanced Lassa fever. Nat. Med..

[13] Plotkin Stanley A. (2017). Vaccines for epidemic infections and the role of CEPI. Human Vaccines & Immunotherapeutics.

[14] Eberhardt Kirsten Alexandra, Mischlinger Johannes, Jordan Sabine, Groger Mirjam, Günther Stephan, Ramharter Michael (2019). Ribavirin for the treatment of Lassa fever: A systematic review and meta-analysis. International Journal of Infectious Diseases.

[15] Okokhere P (2018). Clinical and laboratory predictors of Lassa fever outcome in a dedicated treatment facility in Nigeria: a retrospective, observational cohort study. Lancet Infect. Dis..

[16] Walker DH (1982). Pathologic and virologic study of fatal Lassa fever in man. Am. J. Pathol..

[17] Bausch DG (2001). Lassa fever in Guinea: I. Epidemiology of human disease and clinical observations. Vector Borne Zoonotic Dis..

[18] McCormick JB (1987). A case-control study of the clinical diagnosis and course of Lassa fever. J. Infect. Dis..

[19] Monath TP, Maher M, Casals J, Kissling RE, Cacciapuoti A (1974). Lassa fever in the Eastern Province of Sierra Leone, 1970-1972. II. Clinical observations and virological studies on selected hospital cases. Am. J. Trop. Med. Hyg..

[20] Boisen ML (2015). Multiple circulating infections can mimic the early stages of viral hemorrhagic fevers and possible human exposure to filoviruses in Sierra Leone prior to the 2014 outbreak. Viral Immunol..

[21] Wulff H, Lange JV (1975). Indirect immunofluorescence for the diagnosis of Lassa fever infection. Bull. World Health Organ..

[22] Niklasson BS, Jahrling PB, Peters CJ (1984). Detection of Lassa virus antigens and Lassa virus-specific immunoglobulins G and M by enzyme-linked immunosorbent assay. J. Clin. Microbiol..

[23] Bausch DG (2000). Diagnosis and clinical virology of Lassa fever as evaluated by enzyme-linked immunosorbent assay, indirect fluorescent-antibody test, and virus isolation. J. Clin. Microbiol..

[24] Branco LM (2011). Emerging trends in Lassa fever: redefining the role of immunoglobulin M and inflammation in diagnosing acute infection. Virol. J..

[25] Grove JN (2011). Capacity building permitting comprehensive monitoring of a severe case of Lassa hemorrhagic fever in Sierra Leone with a positive outcome: case report. Virol. J..

[26] Boisen ML (2018). Field validation of recombinant antigen immunoassays for diagnosis of Lassa fever. Sci. Rep..

[27] Ehichioya, D. U. *et al*. Phylogeography of Lassa virus in Nigeria. *J Virol*, 10.1128/jvi.00929-19 (2019). 10.1128/JVI.00929-19 PMC6803284 31413134

[28] Ilori EA (2019). Epidemiologic and clinical features of Lassa fever outbreak in Nigeria, January 1-May 6, 2018. Emerg. Infect. Dis..

[29] Siddle KJ (2018). Genomic Analysis of Lassa Virus during an Increase in Cases in Nigeria in 2018. N. Engl. J. Med..

[30] Kafetzopoulou LE (2019). Metagenomic sequencing at the epicenter of the Nigeria 2018 Lassa fever outbreak. Science.

[31] Branco LM (2008). Bacterial-based systems for expression and purification of recombinant Lassa virus proteins of immunological relevance. Virol. J..

[32] Hastie KM (2016). Crystal structure of the oligomeric form of Lassa virus matrix protein Z. J. Virol..

[33] Trombley AR (2010). Comprehensive panel of real-time TaqMan polymerase chain reaction assays for detection and absolute quantification of filoviruses, arenaviruses, and New World hantaviruses. Am. J. Trop. Med. Hyg..

[34] Nikisins S (2015). International external quality assessment study for molecular detection of Lassa virus. PLoS Negl. Trop. Dis..

[35] Altona. RealStar® Lassa Virus RT-PCR Kits RUO, https://www.altona-diagnostics.com/en/products/reagents-140/reagents/realstar-real-time-pcr-reagents/realstar-lassavirus-rt-pcr-kit-ruo.html (2019).

[36] Branco LM (2011). Lassa hemorrhagic fever in a late term pregnancy from northern Sierra Leone with a positive maternal outcome: case report. Virol. J..

[37] Emperador DM, Yimer SA, Mazzola LT, Norheim G, Kelly-Cirino C (2019). Diagnostic applications for Lassa fever in limited-resource settings. BMJ Glob. Health.

[38] Mazzola LT, Kelly-Cirino C (2019). Diagnostics for Lassa fever virus: a genetically diverse pathogen found in low-resource settings. BMJ Glob. Health.

[39] de La Vega MA (2015). Ebola viral load at diagnosis associates with patient outcome and outbreak evolution. J. Clin. Invest..

[40] Weidmann M (2016). Experiences of outbreak laboratory management in the Ebola Disease outbreak in West-Africa 2014–2015. Clin. Microbiology Infect. Dis..

[41] Safronetz D (2013). Geographic distribution and genetic characterization of Lassa virus in sub-Saharan Mali. PLoS neglected tropical Dis..

[42] Whitmer SLM (2018). New lineage of Lassa virus, Togo, 2016. Emerg. Infect. Dis..

[43] Price ME, Fisher-Hoch SP, Craven RB, McCormick JB (1988). A prospective study of maternal and fetal outcome in acute Lassa fever infection during pregnancy. BMJ.

[44] Okogbenin Sylvanus, Okoeguale Joseph, Akpede George, Colubri Andres, Barnes Kayla G., Mehta Samar, Eifediyi Reuben, Okogbo Felix, Eigbefoh Joseph, Momoh Mojeed, Rafiu Mojeed, Adomeh Donatus, Odia Ikponmwosa, Aire Chris, Atafo Rebecca, Okonofua Martha, Pahlman Meike, Becker-Ziaja Beate, Asogun Danny, Okokhere Peter, Happi Christian, Günther Stephan, Sabeti Pardis C., Ogbaini-Emovon Ephraim (2019). Retrospective Cohort Study of Lassa Fever in Pregnancy, Southern Nigeria. Emerging Infectious Diseases.

[45] McCormick JB (1986). Lassa fever. Effective therapy with ribavirin. N. Engl. J. Med..

[46] Madu IG (2018). A potent Lassa virus antiviral targets an arenavirus virulence determinant. PLoS Pathog..

[47] Drosten C, Panning M, Guenther S, Schmitz H (2002). False-negative results of PCR assay with plasma of patients with severe viral hemorrhagic fever. J. Clin. Microbiol..

[48] Demby AH, Chamberlain J, Brown DW, Clegg CS (1994). Early diagnosis of Lassa fever by reverse transcription-PCR. J. Clin. Microbiol..

[49] Panning M (2010). Laboratory diagnosis of Lassa fever, liberia. Emerg. Infect. Dis..

[50] Wiley Michael R, Fakoli Lawrence, Letizia Andrew G, Welch Stephen R, Ladner Jason T, Prieto Karla, Reyes Daniel, Espy Nicole, Chitty Joseph A, Pratt Catherine B, Di Paola Nicholas, Taweh Fahn, Williams Desmond, Saindon Jon, Davis William G, Patel Ketan, Holland Mitchell, Negrón Daniel, Ströher Ute, Nichol Stuart T, Sozhamannan Shanmuga, Rollin Pierre E, Dogba John, Nyenswah Tolbert, Bolay Fatorma, Albariño César G, Fallah Mosoka, Palacios Gustavo (2019). Lassa virus circulating in Liberia: a retrospective genomic characterisation. The Lancet Infectious Diseases.

[51] Lin WH, Kouyos RD, Adams RJ, Grenfell BT, Griffin DE (2012). Prolonged persistence of measles virus RNA is characteristic of primary infection dynamics. Proc. Natl Acad. Sci. USA.

[52] Simon ID, van Rooijen N, Rose JK (2010). Vesicular stomatitis virus genomic RNA persists *in vivo* in the absence of viral replication. J. Virol..

[53] Bernasconi V (2020). Developing vaccines against epidemic-prone emerging infectious diseases. Bundesgesundheitsblatt, Gesundheitsforschung, Gesundheitsschutz.

[54] Barnes, K. G. *et al*. Deployable CRISPR-Cas13a diagnostic tools to detect and report Ebola and Lassa virus cases in real-time. *submitted* (2020). 10.1038/s41467-020-17994-9 PMC7431545 32807807

[55] Matranga, C. B. *et al*. Unbiased deep sequencing of RNA viruses from clinical samples. *J. Vis.Exp*. **113**, 10.3791/54117 (2016). 10.3791/54117 PMC4993327 27403729

[56] Grabherr MG (2011). Full-length transcriptome assembly from RNA-Seq data without a reference genome. Nat. Biotchnol.

