# Supplementary material for: Field evaluation of a Pan-Lassa rapid diagnostic test during the 2018 Nigerian Lassa fever outbreak
Source: Sci Rep. 2020 May 26;10:8724. doi: 10.1038/s41598-020-65736-0 (PMC7250850; doi:10.1038/s41598-020-65736-0)
Supplement: Supplementary file 1 — Supplementary Information. [file 41598_2020_65736_MOESM1_ESM.docx]

**SUPPLEMENTARY INFORMATION**

**Field evaluation of a Pan-Lassa rapid diagnostic test during the 2018 Nigerian Lassa fever outbreak**

Matthew L. Boisen^1^, Eghosa Uyigue^2,3,4^, John Aiyepada^5^, Katherine J. Siddle^5,6^, Lisa Oestereich^7,8^, Diana K. S. Nelson^1^, Duane J. Bush^1^, Megan M. Rowland^1^, Megan L. Heinrich^1^, Philomena Eromon^2^, Adeyemi T. Kayode^2,3^, Ikponmwosa Odia^4^, Donatus I. Adomeh^4^, Ekene B. Muoebonam^4^, Patience Akhilomen^4^, Grace Okonofua^4^, Blessing Osiemi^4^, Omigie Omoregie^4^, Michael Airende^4^, Agbukor Jacqueline^4^, Ehikhametalor Solomon^4^, Aire Chris Okafi^4^, Sophie Duraffour^7,8^, Meike Pahlmann^7,8^, Kayla G. Barnes^5,9^, Samar Mehta^5,10^, Mambu Momoh^11,12,13^, John Demby Sandi^12,13^, Augustine Goba^12,13^, Onikepe A. Folarin^2,3^, Ephraim Ogbaini-Emovan^4^, Danny A. Asogun^4^, Ekaete A. Tobin^4^, George Akpede^4^, Sylvanus Okogbenin^4^, Peter Okokhere^4,14,15^**,** Donald S. Grant^12,13,16^, John S. Schieffelin^17^, Pardis C. Sabeti^5,6,9,18,19^**,** Stephan Günther^7,8^, Christian T. Happi^2,3,4,9^*, Luis M. Branco^1^***** and Robert F. Garry^1,20,21^*

^1^ Zalgen Labs, LLC, Germantown, MD, USA

^2^The African Center of Excellence for Genomics of Infectious Diseases, Redeemer’s University, Ede, Osun State, Nigeria

^3^Department of Biological Sciences, College of Natural Sciences, Redeemer’s University, Ede, Osun State, Nigeria

^4^Institute of Lassa Fever Research and Control, Irrua Specialist Teaching Hospital, Irrua, Edo State, Nigeria

^5^The Broad Institute of Massachusetts Institute of Technology (MIT) and Harvard University, Cambridge, MA, USA.

^6^The Center for Systems Biology, Department of Organismic and Evolutionary Biology, Harvard University, Cambridge, MA, USA

^7^Bernhard Nocht Institute for Tropical Medicine, Hamburg, Germany

^8^German Center for Infection Research (DZIF), Partner site Hamburg – Lübeck – Borstel – Riems, Germany

^9^Department of Immunology and Infectious Diseases, Harvard T.H. Chan School of Public Health, Harvard University, Boston, Massachusetts 02115, USA.

^10^Beth Israel Deaconess Medical Center, Division of Infectious Diseases, Boston, MA, USA

^11^Eastern Polytechnic Institute, Kenema, Sierra Leone

^12^Viral Hemorrhagic Fever Program, Kenema Government Hospital, Kenema, Sierra Leone

^13^Ministry of Health and Sanitation, Freetown, Sierra Leone

^14^The Department of Medicine, Irrua Specialist Teaching Hospital, Irrua, Edo State, Nigeria

^15^The Department of Medicine, Faculty of Clinical Sciences, Ambrose Alli University, Ekpoma, Edo State, Nigeria

^16^College of Medicine and Allied Health Sciences, University of Sierra Leone, Freetown, Sierra Leone

^17^Sections of Infectious Disease, Departments of Pediatrics and Internal Medicine, School of Medicine, Tulane University, New Orleans, LA, USA

^18^Harvard-MIT Health Sciences and Technology, MIT, Cambridge, MA, USA

^19^Howard Hughes Medical Institute, Chevy Chase, MD, USA

^20^Tulane Health Sciences Center, Tulane University, New Orleans, LA, USA

^21^ Tulane University, School of Medicine, Department of Microbiology and Immunology, New Orleans, LA, USA

***Corresponding Authors**

**Table S1. Case definitions**

| Case definition | Number of subjects | Antigen | IgM | IgG |
| --- | --- | --- | --- | --- |
| Acute Lassa fever | 118 | positive | Either positive or negative | Either positive or negative |
| Post-acute Lassa fever | 115 | negative | positive | Either positive or negative |
| Non-Lassa fever | 197 | negative | negative | Either positive or negative |

**Table S2. Study Database – Acute Lassa fever Cohort**

**
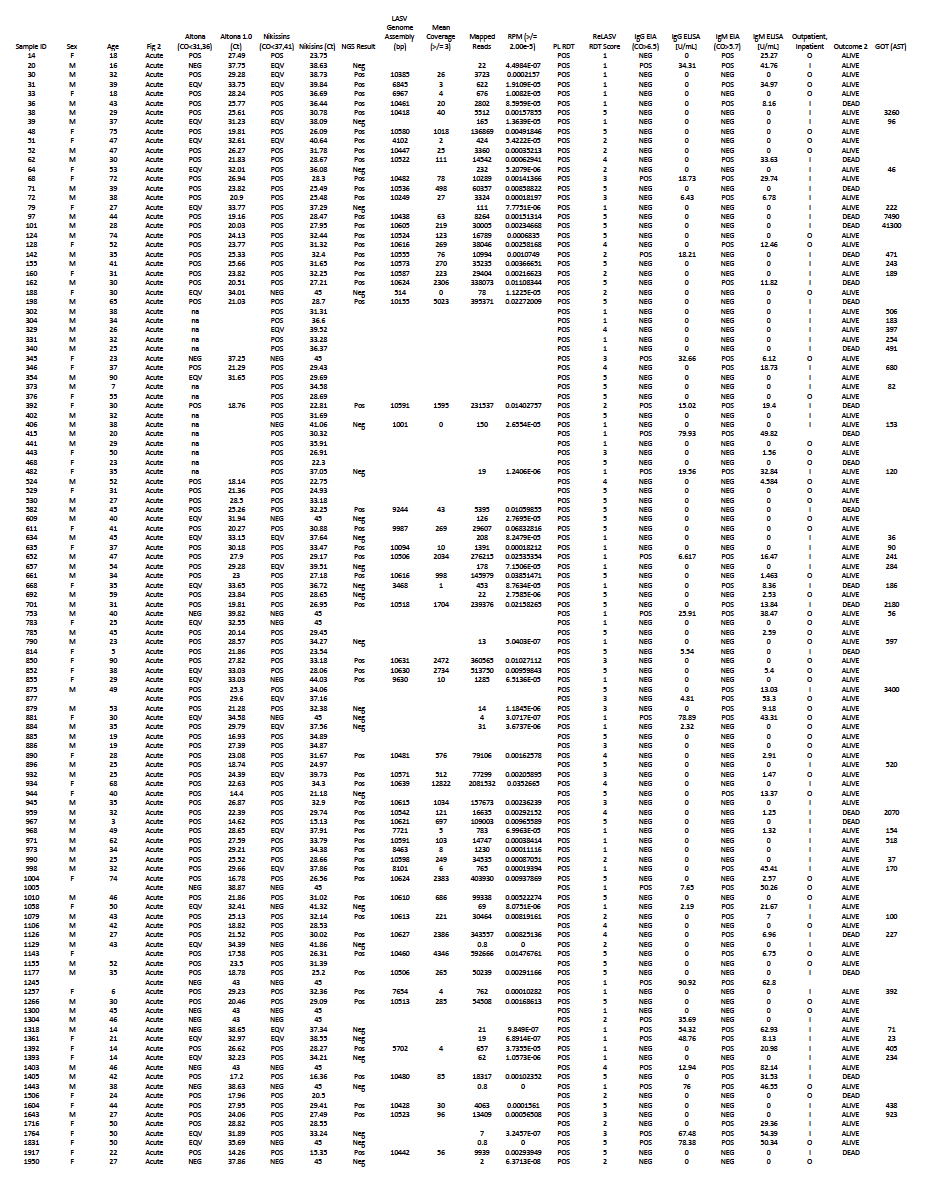
**

**Table S3. Study Database – Post-Acute LF Cohort**

**
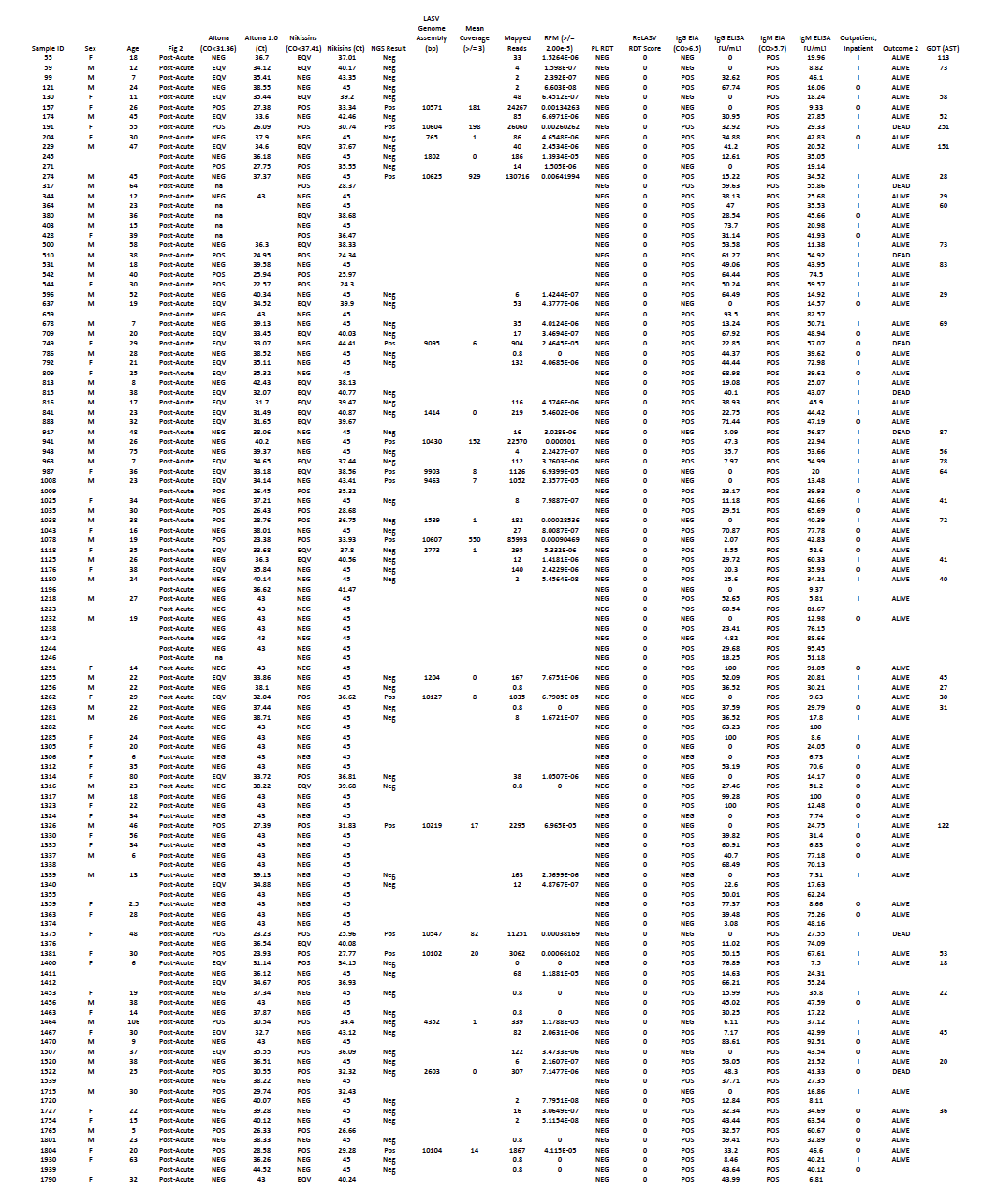
**

**Table S4. Study Database – Non-Lassa Cohort**

**
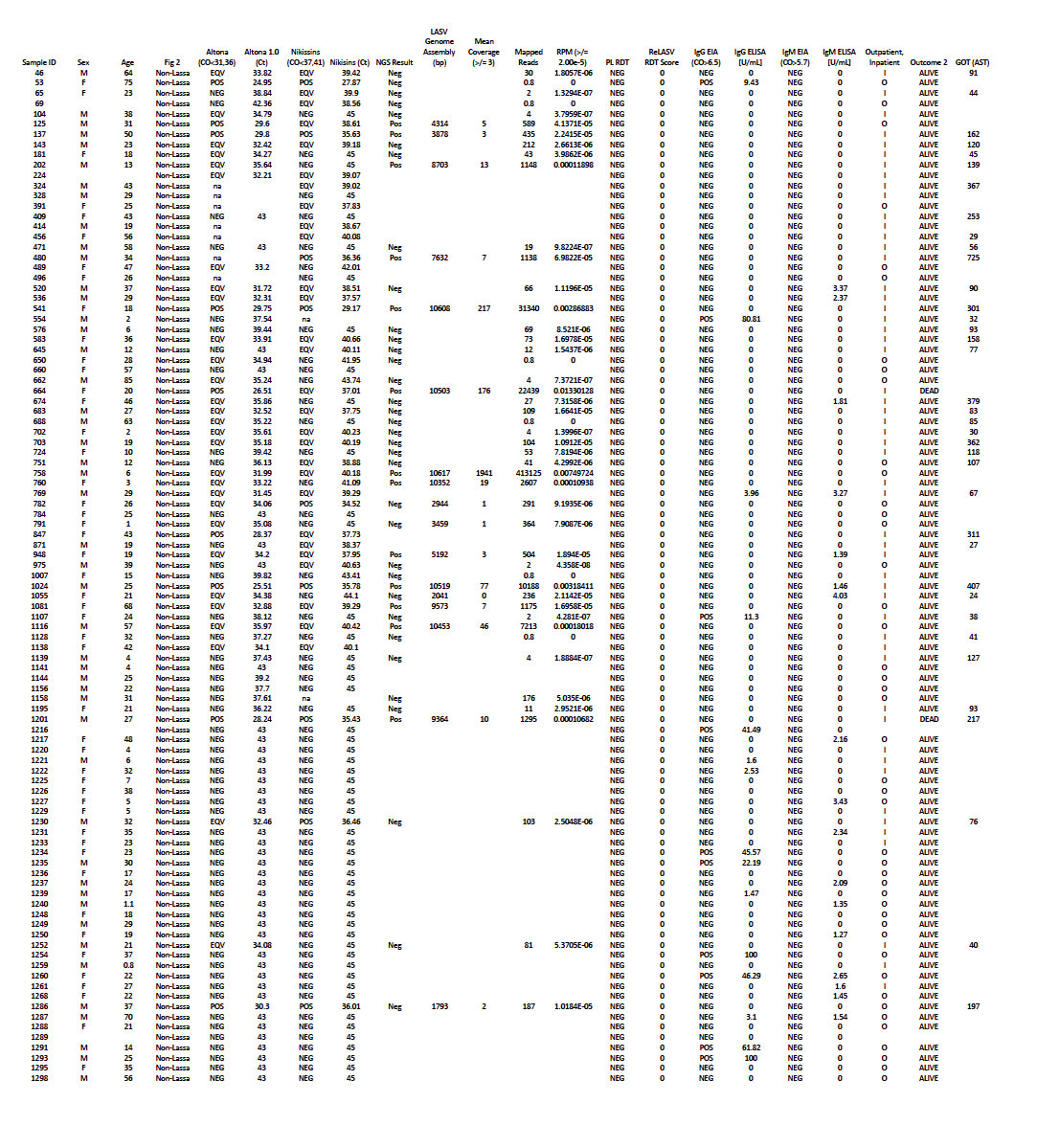
**

**Table S4 Continued. Study Database – Non-Lassa Cohort**

**
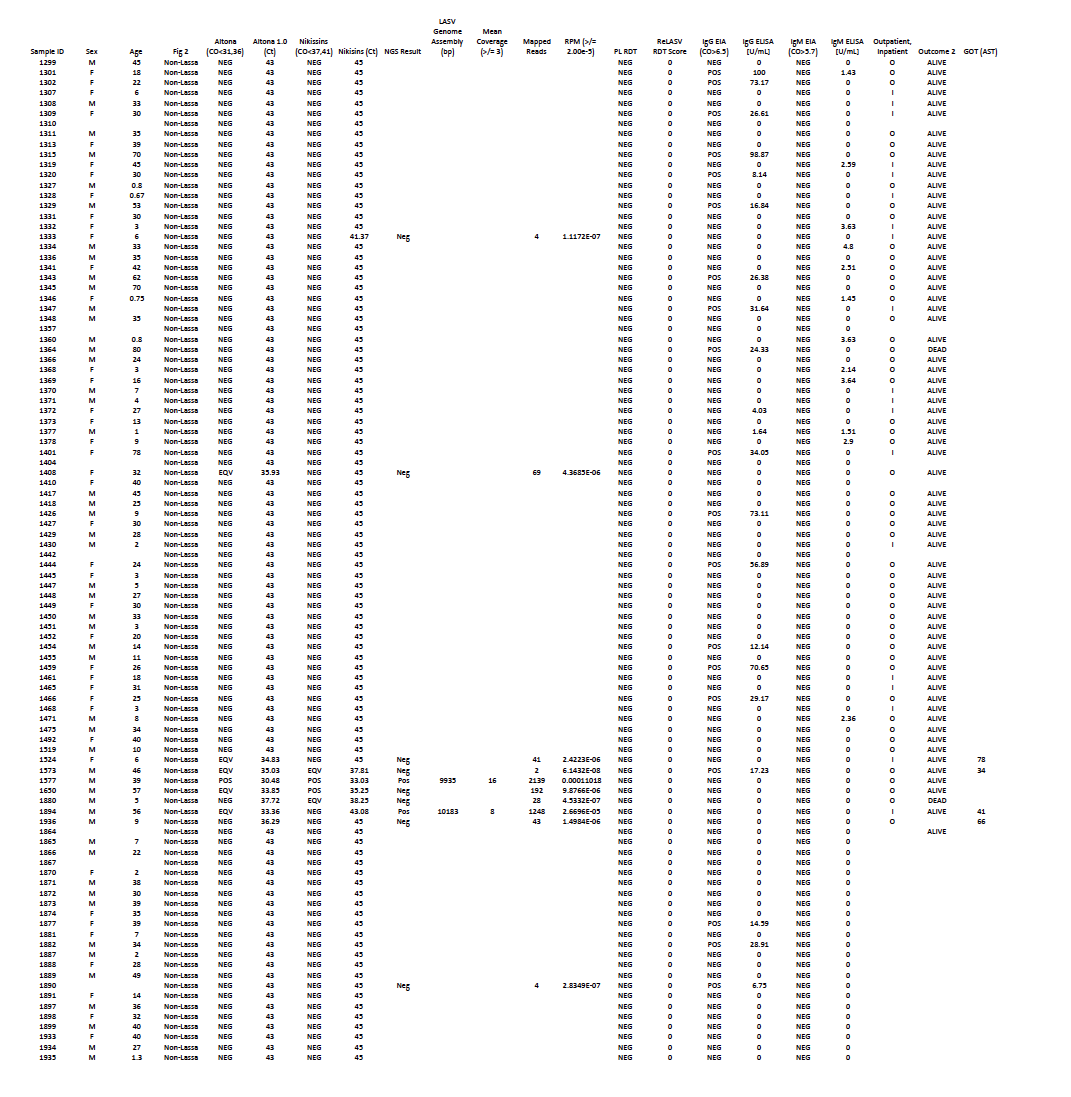
**

Table S5

**Table S6. Altona 1.0 qPCR cut-off ROC analysis**

**Table S7. Nikisins qPCR cut-off ROC analysis**


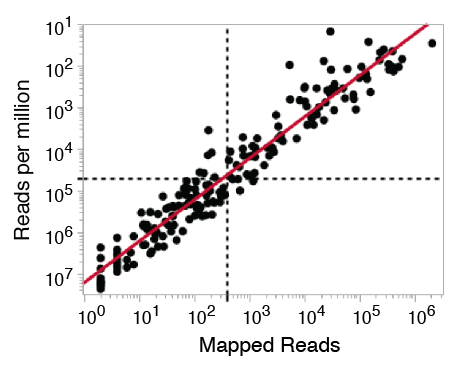


**Figure S1.** Correlation of reads per million versus mapped reads from Next Generation sequencing of Lassa virus genomes (n=182, R^2^ = .93, Linear Regression = -16.62 + 1.00).

**
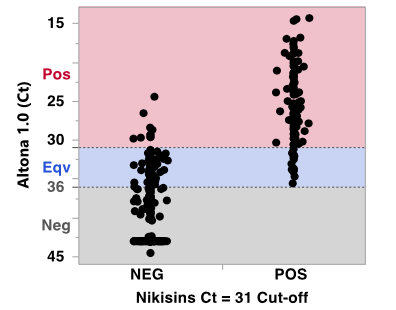
**

**Figure S2. Altona 1.0 Ct Distribution Analysis of Equivocal Range.**

**
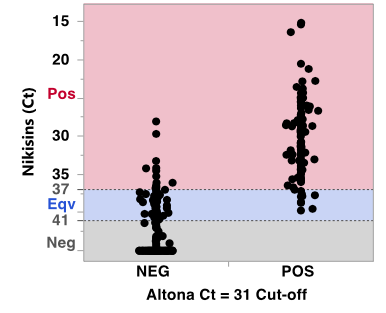
**

**Figure S3. Nikisins Ct Distribution Analysis of Equivocal Range.**

**Figure S4. Correlations of LASV NP Antigen and anti NP-specific IgM or IgG Detection.** Left panel: Pan Lassa RDT visual score versus anti NP-specific IgM (n=430, R^2^=.02, Linear Regression = .94 - .01). Middle panel: Pan Lassa RDT visual score versus anti NP-specific IgG (n=430, R^2^=.04, Linear Regression = .99 - .01). Right panel: anti NP-specific IgM versus anti NP-specific IgG (n=430, R^2^=.30, Linear Regression = 6.52 + .50). ● NP Antigen RDT Positive, ● IgM ELISA Positive, ● IgM and IgG ELISA Positive, ● IgG ELISA Positive, ● ReLASV RDT and G/M ELISA Negative; Black Line – Linear Regression)
